# To what extent are Canadian second language policies evidence-based? Reflections on the intersections of research and policy

**DOI:** 10.3389/fpsyg.2014.00358

**Published:** 2014-05-07

**Authors:** Jim Cummins

**Affiliations:** Department of Curriculum, Teaching and Learning, Ontario Institute for Studies in Education, The University of TorontoToronto, ON, Canada

**Keywords:** core French, French immersion, identity, language policy, multilingualism, second language learning

## Abstract

The paper addresses the intersections between research findings and Canadian educational policies focusing on four major areas: (a) core and immersion programs for the teaching of French to Anglophone students, (b) policies concerning the learning of English and French by students from immigrant backgrounds, (c) heritage language teaching, and (d) the education of Deaf and hard-of hearing students. With respect to the teaching of French, policy-makers have largely ignored the fact that most core French programs produce meager results for the vast majority of students. Only a small proportion of students (<10%) attend more effective alternatives (e.g., French immersion and Intensive French programs). With respect to immigrant-background students, a large majority of teachers and administrators have not had opportunities to access the knowledge base regarding effective instruction for these students nor have they had opportunities for pre-service or in-service professional development regarding effective instructional practices. Educational policies in most jurisdictions have also treated the linguistic resources that children bring to school with, at best, benign neglect. In some cases (e.g., Ontario) school systems have been explicitly prohibited from instituting enrichment bilingual programs that would promote students’ bilingualism and biliteracy. Finally, with respect to Deaf students, policy-makers have ignored overwhelming research on the positive relationship between academic success and the development of proficiency in natural sign languages, preferring instead to leave uncorrected the proposition that acquisition of languages such as American Sign Language by young children (with or without cochlear implants) will impede children’s language and academic development. The paper reviews the kinds of policies, programs, and practices that could be implemented (at no additional cost) if policy-makers and educators pursued evidence-based educational policies.

## THE CANADIAN POLICY CONTEXT

Although Canada enjoys a strong international reputation as a leader in the area of second language teaching, primarily as a result of the implementation of French immersion programs in the 1960s, the development of policies at federal and provincial levels with respect to language teaching has been largely incoherent. Because education falls under provincial jurisdiction, different policies and provisions in relation to language teaching exist in different provinces. All provinces strongly support the learning of the second official language (French in English Canada, and English in Quebec) and they also provide support for newcomer students to learn the language of school instruction but few have developed coherent policies regarding the multilingual realities of schools and communities. Similarly, at the federal level, the 1971 policy of *multiculturalism within a bilingual framework* omitted any meaningful consideration of languages other than the two official languages, substituting positive rhetoric in relation to the *cultural contributions of the other ethnic groups* for any concrete action to foster Canada’s multilingual resources. In the 1970s and 1980s, some funds were provided by the federal government to community groups for purposes of heritage language teaching but those funds were discontinued in the early 1990s. No province has articulated an educational language policy that addresses in a positive way the multilingual realities of its schools, although Alberta at least did consider the issue in the 1980s (Alberta Government, 1988). Some provinces (e.g., Ontario) have articulated restrictive policies in relation to multilingualism by prohibiting use of languages other than English and French as mediums of instruction except on a short-term transitional basis.

The lack of coherence with respect to policy is compounded by the fact that many of the programs and provisions that have been implemented are inconsistent with the empirical evidence regarding effective practice. In this paper, I review this evidence with respect to four spheres of dual language learning: (a) teaching French as a second language (FSL) in English Canada; (b) teaching English and French as additional languages to newcomer students in English Canada and Quebec; (c) teaching heritage languages; and (d) teaching American Sign Language (ASL) to Deaf students in English Canada. I refer to students in these contexts collectively as dual language learners (DLL).

## TEACHING FRENCH AS A SECOND LANGUAGE

### CORE FRENCH PROGRAMS

Core FSL programs typically teach French for 30–40 min each day. Starting grades vary from province to province, and within provinces school boards typically have some discretion regarding the starting grade level. In many parts of Ontario, for example, FSL starts in Grade 4 and continues until at least Grade 9, when it is a compulsory subject for all students.

Results of Core FSL programs have been disappointing. Canadian Parents for French, a federally funded advocacy group, summarized the outcomes as follows: “Only 3% of [Ontario] grade nine core French students continue with the program to Grade 12, most graduating with little ability to converse in, or understand French” (Canadian Parents for French, 2008, p. 17). Research has also shown minimal improvement in students’ French proficiency as a function of length of time in the program. Harley et al. (1988), for example, examined the French proficiency (speaking, listening, reading, and writing) of 574 students in 25 different classes in seven provinces or territories. They found that, with some minor exceptions, performance at the Grade 8 level was unrelated to the starting grade and the length of time the students had been learning French. Few differences were observed regardless of whether students started learning French in Kindergarten, Grade 1, 3, 4, 6, or 8. In other words, one year of Core FSL produced equivalent outcomes to 7+ years, suggesting that core FSL during those years was not particularly effective (see Lapkin et al., 2009, for a more complete review of FSL outcomes).

The persistent failure of Core FSL programs to develop even minimal communicative proficiency in French among a large proportion of participating students highlights the need for a change in policy. There is no empirical support for continuing to pour considerable funds into a program that yields such paltry academic outcomes. Yet, policy-makers across Canada have shown no interest in radically changing Core FSL provision. The ideological commitment to teach both official languages, regardless of the success of this endeavor, trumps the evidence of ineffectiveness.

Much stronger outcomes have been attained both by French immersion and Intensive French (a literacy-oriented half-year immersion in French starting at the Grade 6 level, Netten and Germain, 2005). Extended French programs, where one or more subjects are taught through French (similar to content and language integrated learning, CLIL programs), have also shown much more promising outcomes than Core FSL. However, more than 90% of students studying French in Canada are enrolled in Core FSL rather than in one of these more successful alternatives.

Another program that has demonstrated much more promising outcomes than traditional Core French is the accelerated integrative method (AIM) developed by Canadian educator Wendy Maxwell. AIM is a form of Core French insofar as it is taught for approximately 30–40 min per day but its pedagogical assumptions and outcomes are radically different. AIM has been implemented in more than 4,000 schools across Canada as well as in some international contexts (Arnott, 2011).

### ACCELERATED INTEGRATIVE METHOD

Accelerated integrative method adopts a very different approach to the scope and sequence of L2 teaching than the typical Core French program, which has traditionally been organized around themes (e.g., going to the supermarket, animals in the zoo, etc.). In contrast, AIM integrates the teaching of French with the arts (e.g., drama) and literacy (extensive reading and writing in addition to speaking and listening). AIM scaffolds meaning initially through gestures which enable students to understand full sentences in which each word is represented by a gesture. Basic grammatical markers (e.g., masculine, feminine, past tense, etc.) are also represented by gestures. Reading skills are developed through the use of big books read to the class and supported by gestures. These stories are dramatized by the teacher and students together. Finally, writing is developed after students have mastered the story. Students answer comprehension questions in writing and, with increasing proficiency, script their own dramatizations on the basis of the story.

Maxwell (2004) describes the approach as follows:

Through this approach, all target vocabulary to be learned by the student is taught kinesthetically, visually, and in an auditory manner, thus responding to a variety of learning styles. Because words are kinesthetically represented through gesture, and contextualized through story and drama, students learn to see and feel the language.… Fluency is built by systematically scaffolding the presentation of new vocabulary, beginning with words of highest frequency and widest scope. Words targeted for presentation through gesture and story in this program have been selected … according to frequency, function and ease of acquisition. This target vocabulary, termed pred down language (PDL), places a high emphasis on verbs, but also includes other vocabulary and structures important for beginning fluency development^1^.

Arnott (2011, p. 157) points out that research carried out on the effectiveness of AIM has shown positive results in a number of small scale studies “while larger-scale quantitative and mixed method research suggested that merely using AIM does not make students significantly more proficient in French (Bourdages and Vignola, 2009; Mady et al., 2009).” Her own research focused on exploring the ways in which teachers implemented AIM rather than attempting to compare AIM and non-AIM methods. She points out that comparison of methods is problematic because of significant variation among teachers in the ways in which they implement particular methods.

This reality is evident in the research which failed to find differences between AIM and non-AIM classrooms. For example, in the Mady et al. (2009) study, which reported no differences in French proficiency between AIM and non-AIM Grade eight classrooms, characteristics of the AIM methodology were found in classrooms designated as both AIM and non-AIM. The authors point out: “Observation data used for selecting the sample (Mady et al., 2007) suggest that… characteristics deemed central to AIM were not exclusive to AIM classrooms” (Mady et al., 2009, p. 716). Teachers in each group had attended some AIM training sessions and were using some AIM materials in their classrooms. Thus, the comparison is not particularly robust in assessing the impact of AIM.

The findings of the Bourdages and Vignola (2009) study are also interpreted in very problematic ways by the authors. They interpreted their results as showing “few significant differences between the AIM group and the non-AIM group” (p. 731) in oral interviews conducted with 18 AIM and 16 non-AIM Grade 3 students. In actual fact, there are highly significant differences between the groups in the amount of oral language students produced in French and in the extent to which the students were capable of using French exclusively in the interviews rather than reverting to English. Specifically, the AIM students produced 1751 utterances compared to 811 for the non-AIM students – more than twice as much. The AIM students also produced 1662 utterances completely in French (95%) compared to 306 for the non-AIM group (38%). It is worth noting that teachers of both AIM and non-AIM groups used French exclusively in their instruction so these huge differences cannot be attributed to differential exposure to French.

The authors’ claim that there were few differences between the groups is based on the percentages of utterances produced by each group that had various types of grammatical errors (e.g., gender, verb agreement, etc.). The authors chose to focus on the fact that both groups of early stage learners were making similar grammatical errors rather than on the fact that AIM students demonstrated much greater fluency in French and ability to continue speaking French rather than revert to English when attempting to express themselves. The logic entailed in the conclusions of the Bourdages and Vignola (2009) study is equivalent to claiming that there are no differences in French proficiency between a student who produced more than 20 utterances in the interview, the vast majority of which were in French only, compared to a student who produced only 10 utterances, only four of which were exclusively in French, just because a similar proportion of utterances of each student contained errors of various kinds.

In short, the Bourdages and Vignola (2009) study, contrary to the claims of its authors, provides strong support for the findings of smaller-scale studies showing that the AIM methodology can significantly increase students’ fluency in French. As Arnott (2011) points out, it is not possible to identify which components of AIM are most effective in scaffolding comprehension and production of French but there is clearly a case to be made for incorporating elements of AIM into both Core FSL and French immersion programs.

### FRENCH IMMERSION PROGRAMS

A common finding from L2 immersion programs across a variety of contexts is that students gain a reasonable level of fluency and literacy in L2 at no apparent cost to their academic skills in the socially dominant language. In the Canadian French immersion context, students catch up in most aspects of English standardized test performance within a year of the introduction of formal English language arts. With respect to French skills, students’ receptive skills in French are better developed (in relation to native speaker norms) than are their expressive skills. By the end of elementary school (Grade 6, age 12) students are close to the level of native speakers in understanding and reading of formal French (assessed by standardized tests), but there are significant gaps between them and native speakers in spoken and written French. The gap is particularly evident with respect to accuracy of grammar and range of vocabulary knowledge and use.

These gaps are clearly related to the restricted input that students receive in French. Typically students experience little contact or interaction with French outside the school context. Very few students watch French television or read for pleasure in French. After the initial grades, reading in French tends to be primarily textbook reading, which is typically not particularly engaging for students. Thus, there are few opportunities for students to extend their exposure to French and expand their vocabulary knowledge and grammatical command. Writing also tends to be carried out only within the school context and applied to academic tasks that are often not highly engaging for students.

Despite the fact that there is overwhelming evidence for strong relationships between the development of academic skills in French and English (e.g., Cummins, 2001), there has been little attempt within French immersion programs to teach for transfer across languages. This is because monolingual instructional assumptions have dominated practice within immersion. The rationale for developing bilingualism by means of monolingual instruction was clearly expressed by Lambert (1984, p. 13):

No bilingual skills are required of the teacher, who plays the role of a monolingual in the target language... and who never switches languages, reviews materials in the other language, or otherwise uses the child’s native language in teacher-pupil interactions. In immersion programs, therefore, bilingualism is developed through two separate monolingual instructional routes.

Since the time of the initial St. Lambert program some aspects of the strict separation of languages have become somewhat more relaxed. For example, the same teacher frequently teaches both the French and English parts of the day in Grades 4 through 6. However, the principle of linguistic separation and a total ban on any kind of translation across languages remains largely unchallenged within French immersion theory and practice. I have termed this the *two solitudes assumption* and highlighted its problematic instructional consequences (Cummins, 2007). Among these consequences are (a) the inability to draw students’ attention to the many cognate relationships between French and English, (b) inability to enable students to create and web-publish dual language books that might showcase students’ emerging bilingual skills, (c) inability to pursue partner class projects with French L1 students who are learning English in which the Internet is used to connect learners of each language. The “two solitudes” assumption also discourages educators from coordinated planning that would integrate curriculum objectives in French and English. For example, in teaching writing in French and English, the rules and conventions for paragraph formation could be taught at a similar time in French and English language arts, thereby reinforcing the learning of this content (see Ó Duibhir and Cummins, 2012).

Is there any empirical evidence supporting the two solitudes assumption? During the 50 years that French immersion programs have been in existence, researchers have found no evidence to support this assumption. Even researchers who have been in the forefront of French immersion program evaluations during the past 40 years have advocated more instructional flexibility with respect to bringing the two languages into productive contact (e.g., Swain and Lapkin, 2000, 2013). As a result of the two solitudes assumption, immersion programs have needlessly avoided teaching for transfer across languages and at least some of the limitations observed in students’ French proficiency can be attributed to the failure to exploit the learning efficiencies afforded by bringing the two languages into productive contact. Initial research exploring instructional approaches that promote transfer of morphological and broader literacy skills across French and English in the Canadian context has produced promising results (Lyster et al., 2009, 2013).

In summary, a variety of gaps between research evidence and instructional policies and practices are evident with respect to the teaching of French in Canada. These gaps apply to both Core FSL and to French immersion.

## TEACHING ENGLISH AND FRENCH TO NEWCOMER STUDENTS

A synthesis of research findings from Montreal, Toronto and Vancouver demonstrates that, in general, DLL students from immigrant backgrounds tend to perform relatively well in Canadian schools (McAndrew et al., 2009). However, this apparent success masks considerable variation in DLL students’ academic outcomes. Studies in Alberta (Derwing et al., 1999; Watt and Roessingh, 1994, 2001) revealed that large proportions of DLL students failed to graduate with a high school diploma (60% in the Derwing et al. (1999) study and 74% in the Watt and Roessingh, 1994 study). More recent studies from British Colombia also show a high “disappearance” or non-completion rate among DLL high school students (Gunderson, 2007; Toohey and Derwing, 2008). Immigrant students from higher socioeconomic backgrounds tended to perform considerably better than those from refugee and/or low socioeconomic backgrounds.

Although a stable infrastructure for providing language support services to newcomer students has been established in most provinces, there remain significant gaps in the extent to which educational policies and practices conform to what is implied by the research evidence. For example, there has been a lack of serious policy consideration at all levels of the educational system (provincial ministries, school boards, university-level teacher education programs, and individual schools) regarding the pedagogical implications and opportunities of linguistic diversity. Home languages other than English or French are still viewed by many educators as largely irrelevant to children’s schooling. Consequently, many schools do not encourage bilingual students to showcase their linguistic accomplishments, thereby missing an important opportunity both to enable students to use their L1 as a cognitive tool and develop their L1 abilities to the level of literate competence.

Most classroom teachers at the elementary level and content teachers at the secondary level have had no pre-service or professional development preparation focused on appropriate instruction for DLL students. Educational policies and structures (e.g., teacher education) across Canada have articulated no expectation or requirement that *mainstream* teachers should have any knowledge regarding appropriate ways of scaffolding instruction for second language learners in their classrooms. The implicit assumption in English Canada has been that ESL teachers will take care of “fixing” the language problems of English language learners. This assumption ignores the fact that typically at least five years are required for DLL students to catch up academically in the school language (e.g., Cummins, 1981; Klesmer, 1994). Thus, content teachers will inevitably be teaching DLL students over the course of several years while students are still in the process of catching up to grade expectations in academic English (or French). In an education context characterized by linguistic diversity and high rates of immigration, it is no longer sufficient to be an excellent Science or Mathematics (or other content areas) teacher in a generic sense; excellence must be defined by how well a teacher can teach Science or Mathematics to the students who are in his or her classroom, many of whom may be in the early or intermediate stages of English (or French in Quebec) language acquisition. Toohey and Derwing (2008), similarly highlight the “untenable situation” whereby “many ESL learners are now taught by teachers who have no training at all in second-language education techniques and approaches” (p. 190).

There is also no articulated expectation that school principals and vice-principals should know anything about appropriate instruction for DLL students from immigrant backgrounds. Principals’ courses typically include no content relating to effective leadership in linguistically diverse schools. Furthermore, the decision-making process within school boards regarding promotion to administrative positions rarely takes account of an individual’s ability to provide instructional leadership in schools with large numbers of linguistically diverse students. One of the duties of administrators in schools is to inspect teachers at regular intervals to ensure that they are delivering effective instruction. If the principal or vice-principal has little awareness of appropriate scaffolding strategies to support DLL students in understanding instruction, how can they assess the extent to which teachers are implementing these strategies effectively?

Solutions to this issue are surprisingly simple and cost-effective. Any school system that wanted to build its capacity to teach effectively in a linguistically diverse context could implement two “no-cost” initiatives that would quickly generate results. First, they could publicly specify the knowledge and expertise they expect of all new teachers they are planning to hire. For example, they could articulate the expectation that all teachers should know how to teach their content areas effectively to students who are in the early and later stages of acquiring English (or French in Quebec). They could also specify that content teachers should know how to articulate and teach linguistic objectives as well as content objectives in their teaching practice. The announcement of this initiative could also include a sample of the kinds of questions regarding appropriate instruction for DLL students that applicants for teaching positions could expect to be asked. These policies would put immediate pressure on Faculties of Education to ensure that new teachers have the opportunity to develop this expertise.

Second, school systems characterized by linguistic diversity could institute criteria for advancement within the school system (e.g., to principal or vice-principal positions) that would *explicitly* require either formal qualifications in ESL or demonstrated expertise in issues related to effective instruction of linguistically diverse students. Specific questions regarding these issues should be asked in interviews for appointment or advancement. For example, school systems might specify that school leaders should be familiar with the core knowledge base regarding (a) trajectories of school language acquisition among newcomer students, (b) the positive role of students’ L1 in facilitating L2 development, (c) instructional strategies (e.g., scaffolding) required to teach academic content effectively to students who are in the process of developing academic English proficiency.

The reluctance of most school systems across Canada to even discuss, let alone institute such policies, together with the inertia that has characterized most Faculties of Education with respect to preparing teachers to teach DLL students, is inconsistent with the commitment to equity and social justice that these institutions claim to endorse.

## TEACHING HERITAGE LANGUAGES

As it has been used in the Canadian context, the term *heritage languages* usually refers to all languages other than the two official languages (English and French), the languages of First Nations (Native) and Inuit peoples, and the languages of the Deaf community (ASL and langue des signes québécoise, LSQ). The term *heritage languages* came into widespread use in 1977 with the establishment of the Heritage Languages Program in the province of Ontario. Funded by the provincial government, this program provides support for the teaching of heritage languages for up to two-and-one-half hours per week outside of the regular 5-hour school day. All students can enroll in these programs regardless of the specific language spoken at home. In the early 1990s, the term heritage languages was changed to international languages by the Ontario provincial government, reflecting misgivings among ethnocultural communities that the notion of “heritage” entailed connotations of learning about past traditions rather than acquiring language skills that have significance for children’s overall educational and personal development. In western Canadian provinces, the term *international* languages is commonly used to refer to languages taught within the public school system (either as subjects of instruction or through bilingual programs) while the term *heritage* languages usually refers to languages taught in programs organized by ethnocultural communities. The terms *heritage* and *international* languages are used interchangeably in the present article.

In Quebec, the government provides funding for the *programme d’enseignement des langues d’origine* (PELO), which was originally introduced in 1977. The rationale for PELO has gone beyond simply promoting skills in students’ home languages; PELO is currently seen by school boards and the Quebec government as a stimulus to enable students to transfer knowledge and skills from one language to the other and from one culture to the other, thereby supporting students in learning French and succeeding academically.

Considerably more openness to the use of heritage/international languages as mediums of instruction is evident in the western Canadian provinces (Alberta, British Colombia, Manitoba, and Saskatchewan) than in eastern Canadian provinces. Bilingual programs involving heritage/international languages exist in all four western provinces. As noted in a previous section, Alberta has been a leader in actively supporting the establishment of bilingual programs in a variety of languages. In 1971, Alberta became the first province to legalize languages other than English or French as mediums of instruction in the public school system. An amendment to the Education Act stated that a “board may authorize (a) that French be used as a language of instruction, or (b) that any other language be used as a language of instruction, in addition to the English language, in any or all of its schools” (Aunger, 2004). In 1973, the Edmonton Public School Board introduced the English-Ukrainian program at the Kindergarten level and an English-German program followed in the fall of 1978. Currently, Alberta offers 50/50 English/heritage language bilingual programs in ASL, Arabic, German, Hebrew, Mandarin, Polish, Spanish, and Ukrainian. The Spanish program has grown significantly in recent years and currently serves more than 3,000 students. First Nations Band-operated bilingual programs are also offered in Blackfoot and Cree (see Alberta Government, 2006).

It is interesting to relate the teaching of international languages to the teaching of French discussed in an earlier section. The international language bilingual programs have been evaluated as highly successful in developing moderately strong heritage language skills at no cost to students’ English proficiency (see Cummins and Danesi, 1990, for a review). In this respect they parallel the outcomes of French immersion and CLIL programs. No formal evaluation has been carried out on heritage language programs taught as a subject outside the school day but indications are that both the quality of teaching and outcomes are mixed (Cummins and Danesi, 1990). This is not surprising in view of the limited success of Core FSL programs which have much higher status and institutional support.

Thus only the western provinces (particularly Alberta) have implemented evidence-based programs to support the teaching of heritage languages. In Ontario, as noted in a previous section, it is illegal to teach through the medium of a heritage language except on a short-term transitional basis to help students learn English. It is instructive to examine the reasoning of the Royal Commission on Learning (1994) which considered this issue in its report. The Commissioners acknowledged the range of submissions they received supporting an amendment to the Education Act to permit heritage languages to be used as mediums of instruction and they also acknowledged that enrichment bilingual programs were in operation in several other provinces. However, they went on to note:

We do not recommend a change in Ontario’s legislation with respect to languages of instruction at this time. We strongly support the use of other languages as a transitional strategy, which is already permitted... We also support a learning system that places more value on languages as subjects, and we hope that many more students will learn third (and fourth) languages, and take courses in them at secondary and post-secondary levels.... But we are very concerned that all students in Ontario be truly literate in one of the official languages. In our view, the school system is obliged to help students function at a high level in English or French, and to gain a reasonable knowledge of the other official language. We appreciate the value of the existing, optional International- (formerly Heritage-) Language program, elementary, but we are not prepared to go well beyond that by suggesting that students be educated in an immersion or bilingual program in any one of a vast number of non-official languages (Royal Commission on Learning, 1994, pp. 106–107).

The Commissioners’ failure to engage with the research evidence on this issue is, unfortunately, very obvious. They imply that students who enroll in a bilingual program involving English and a heritage language (such as the Alberta programs outlined above) will fail to become “truly literate” in English or French despite the fact that there is not a shred of evidence from the Alberta programs or any other bilingual program for minority group students to support this assumption (Cummins and Danesi, 1990). They raise the specter of demands for bilingual programs from speakers of a “vast number of non-official languages” despite the fact that the demand for heritage language bilingual programs in both the Prairie provinces and in Ontario has been modest.

In summary, with the notable exception of the province of Alberta, and to a lesser extent the other western provinces, Canadian provinces have shown little interest in imaginative approaches to heritage language education. Because there has been little sustained demand from ethnocultural communities to implement bilingual programs, governments have stood on the sidelines and declined to show any leadership regarding the promotion of Canada’s linguistic resources.

## DENYING DEAF CHILDREN BILINGUAL OPPORTUNITIES

Several phases can be identified in the history of Deaf children’s education. The first phase emerged from the initial founding of a school for Deaf students by Abbé de l’Epée in Paris, France in 1760. As pointed out by Gibson et al. (1997), “a natural outgrowth was the emergence of a Deaf community, the essential circumstance in which a language – sign language – could develop” (p. 231). In the early 1800s Thomas Gallaudet, an American educator, went to Paris to learn more about the methods of educating Deaf students that had been developed in the French context. Later, he returned to the United States and, with Laurent Clerc, a Deaf master teacher from the Paris school, founded the first school for Deaf students in the United States in 1817. ASL evolved as the French sign language used by Clerc merged with the sign language used by local Deaf people.

What many Deaf communities regard as the “Golden Age” of Deaf education ended with the adoption of an exclusively oral instructional approach by delegates at the 1880 International Congress of Educators of the Deaf in Milan, Italy. This approach dominated the education of the Deaf for almost 100 years and continues to be implemented in a shrinking number of schools internationally. The auditory/oral approach emphasizes the development of any residual hearing with the assistance of hearing aids and the development of speech-reading skills and speech production. A major part of the rationale for an exclusive reliance on the auditory/oral modality was that children will not make the effort to develop oral language if they are permitted to use the “crutch” of sign language.

Gibson et al. (1997) point out that by the early 1970s, educators began to realize “the disastrous effects the oral, monolingual approach had on the spoken and written English of the students, many of whom graduated from oral programs illiterate in both ASL and English” (p. 232). Swanwick (2010) similarly notes that research in the United Kingdom and elsewhere showed that “deaf pupils left school with median reading ages of nine; with poor speech intelligibility and with lip-reading skills no better than those of the hearing population, despite focused training in this” (p. 149).

The Total Communication approach began to replace an exclusively auditory/oral approach during the 1970s. This approach involves the simultaneous use of spoken language together with a signed form of the spoken language. These signed forms of spoken languages have been controversial both among educators and Deaf communities in many countries. In some countries many members of the Deaf community use a signed form of the spoken language but in others (e.g., Canada) a significant proportion of the Deaf community rejects this form of manually coded language as an artificial imposition from hearing educators and policy-makers.

This skepticism in relation to the effectiveness of Total Communication approaches is reinforced as a result of the fact that these programs have failed to increase the academic achievement of Deaf students in any significant way. As pointed out by Allen (1986, p. v): “After 25 years of Total Communication the average deaf high school graduate had achieved a third to fourth grade level education (Allen, 1986).”

As a result of the failure of Total Communication approaches, debate has shifted to the feasibility and rationale for implementing bilingual/bicultural approaches that would use a natural sign language together with the dominant spoken/written language as mediums of instruction. Initial implementation of bilingual instructional approaches took place in Sweden in the early 1980s and bilingual programs have spread to other contexts (e.g., in Europe, North American, and Japan) since that time. However, there remain many areas of controversy with respect to the theoretical and empirical rationale for bilingual/bicultural programs and the appropriate ways of implementing them. For example, within North America and elsewhere there is debate about whether the development of ASL fluency might impede spoken English acquisition among Deaf children who have received cochlear implants. There is also the question as to whether cross-lingual transfer will occur between a manual signed language and a spoken/written language in the same way that it does between two spoken/written languages.

Research provides a definitive answer to this latter question. Many studies (reviewed by Hermans et al., 2010; Cummins, 2011) have consistently demonstrated significant relationships between students’ proficiency in ASL and their development of English reading and writing skills. Transfer between sign language and written/spoken language has been reported at lexical, morphological, syntactic, and pragmatic levels (e.g., Padden and Ramsey, 1998; Menéndez, 2010). These positive relationships can be attributed to transfer of conceptual elements (knowledge of the world) across languages, transfer of metacognitive and metalinguistic elements, and some specific linguistic elements (e.g., fingerspelling, initialized signs).

To what extent does this pattern hold for Deaf children who have undergone cochlear implants? This question is important because currently in Ontario and most other parts of Canada, children who receive cochlear implants are required to forgo the opportunity to learn ASL/LSQ if they wish to receive audio/verbal therapy (AVT), considered necessary to train the brain to hear and comprehend spoken language. AVT professionals mandate that children receiving AVT not acquire ASL and they discontinue the program if children are exposed to ASL input or instruction. Their rationale is that ASL will interfere with children’s ability and motivation to acquire speech by causing auditory areas of the brain to be reallocated to visual processing. As Snoddon (2008) points out, there is absolutely no evidence to support this policy. In fact, although research on the issue is limited, the existing evidence supports the development of bilingualism (e.g., ASL/English) among students who have received cochlear implants. In Swedish research (Preisler et al., 2002) children with cochlear implants who had developed fluency in Swedish sign language showed better speech production than similar students who had not developed sign language fluency. This is consistent with the more general research on ASL/English relationships showing positive transfer across languages. In contrast to Canada, Sweden strongly encourages children who receive cochlear implants to acquire Swedish sign language (Preisler and Ahlström, 1997; Bagga-Gupta, 2004).

In short, once again we find a language education context in Canada where evidence-free assumptions rather than research findings determine policy and practice. Not only are children who receive cochlear implants denied the opportunity to develop bilingualism, crucial time during their early years is spent learning how to decode speech instead of engaging in genuine communication that develops concepts and expands their minds.

## SOME POSITIVE DEVELOPMENTS

Although the discussion to this point has focused on *gaps* between the research evidence and Canadian policies and practice in language education, some emerging positive directions should be noted. Across Canada, a series of collaborations between educators and university researchers has begun to explore two orientations which we (Cummins and Persad, in press) have termed (a) *teaching through an EAL (English-as-an-additional-language) lens* and (b) *teaching through a multilingual lens*. These complementary orientations to pedagogy reflect school-based language policies that articulate all teachers’ responsibility to provide effective and comprehensible instruction across the curriculum to DLL students rather than view this role only as the responsibility of the language specialist teacher. They also articulate the opportunities that all teachers have to expand students’ language awareness and expertise in the context of subject matter instruction. Thus, at the secondary level, the science teacher would see herself not only as a teacher of science but also a teacher of the language of science. This implies that she articulates language objectives as well as content objectives in her lesson plans.

Teaching through a multilingual lens incorporates the philosophy and pedagogical practices of teaching through an EAL lens but broadens the pedagogical orientation to position students’ multilingual abilities as personal, cognitive, and academic resources for learning. Teachers explicitly orient their instruction to promote two-way transfer across languages and communicate to students that their language talents represent intellectual accomplishments that are valued by the school and, by implication, the wider society. Thus, teachers actively challenge the devaluation of Canada’s multilingual resources that is implicit in the intellectual inertia of policy-makers and educational leaders in relation to this issue. Imaginative leadership from administrators is essential for the school to move in a coordinated and coherent way in the direction of teaching through EAL and multilingual lens.

The principles underlying teaching through EAL and multilingual lens have been articulated in a variety of ongoing projects that have documented the classroom implementation and outcomes of concrete instructional strategies (e.g., Armand et al., 2008; Dagenais et al., 2008a,b; Marshall and Toohey, 2010; Cummins and Early, 2011; Lotherington, 2011; Roessingh, 2011; Chumak-Horbatsch, 2012; Naqvi et al., 2012; Stille and Cummins, 2013; Ntelioglou et al., submitted). Rather than attempting to review these projects in any detail (see Cummins and Persad, in press), I will simply list the kinds of classroom activities that are implied by these pedagogical orientations. Four categories of activity or project work are described, ranging from the very simple to the more elaborate. It is noteworthy that implementation of these projects requires no additional financial or material resources; they simply entail some instructional imagination and a commitment to teach the whole child.

### SIMPLE EVERYDAY PRACTICES TO MAKE STUDENTS’ LANGUAGES VISIBLE AND AUDIBLE WITHIN THE SCHOOL

• Each day, one or two students bring a word from their languages into the classroom and explain why they chose that word and what it means. All students and the teacher learn that word. The multilingual words that the class has learned can be displayed on a word wall that rotates every month. The words can also be included into a computer file that can be printed out on a regular basis for review by students and teachers.
• All students including the teacher learn simple greetings (hello, thank you, etc.) in the languages of the classroom. Students who speak these languages are the “teachers.” The “teachers” can also show their peers and teacher how to write a few simple expressions in different scripts (e.g., Arabic, Chinese, Greek, etc.).
• During the morning announcements, students give greetings and say a few words in different languages (with follow-up translation in English).
• At school assemblies, teachers who speak additional languages (including French) say a few words in a language other than English and a student also gives greetings in a language other than English.
• Examples of students’ work in English and L1 are prominently displayed in school corridors and at the entrance to the school in order to reinforce the message to parents and students that students’ linguistic talents are seen as educational and personal assets within the school.

These simple activities have the potential to sensitize students to the sounds and writing systems of different languages and counteract the ambivalence and even shame that many students develop in relation to their languages. The unveiling of students’ languages within the classroom can also be linked to other curricular content. For example, if a Sri Lankan Tamil student has brought a word from her language to share with the teacher and her classmates, this could be extended to demonstrating where Sri Lanka is on a map of the world and explaining some salient aspects of its culture and history.

### ENCOURAGING STUDENTS TO USE THEIR L1s FOR READING, RESEARCH, NOTE-TAKING ETC.

• Beyond the early grades, newcomer and bilingual students could be encouraged to activate their background knowledge of content (e.g., science content) and expand that knowledge by accessing L1 resources that might be available on the Internet (e.g., researching the concept of *photosynthesis* in L1). Building up this L1 knowledge will make L2 content and texts more comprehensible and promote two-way transfer across languages.
• Encourage DLL students to use L1 for group planning of projects which will be presented to the wider class in English. In these cases, L1 is used as a stepping stone to better performance in English where limited English skills do not impede students’ ability to engage with the project.
• Encourage parents (and/or students) to read and/or tell stories in L1 in the home both as a means of expanding L1 knowledge into literate spheres and also expanding knowledge of the world.
• Ensure that the school library has a good collection of L1 and dual language books for students to read and parents to check out for reading at home.
• Invite community members to come to class to read and/or tell stories in community languages (see Naqvi et al., 2012).
• In social studies at intermediate or high school levels, encourage students to research issues and current affairs using Internet sources in their L1s. Parents can assist in this process. Students then bring this information back to class and differences in perspectives across different languages, cultures, and ideologies can be discussed.

### USING TECHNOLOGY IN CREATIVE WAYS TO BUILD AWARENESS OF LANGUAGE, GEOGRAPHY, AND INTERCULTURAL REALITIES

• Google Translate^2^ can be used for a wide variety of purposes – for example, to aid in the “language teaching” outlined above or to assist newcomer students in creating dual language books or projects. For example, students write in L1 and then use Google Translate to generate a rough version in English. The teacher and/or other students can then help the newcomer student edit this rough version into coherent English prose.
• Google Earth can be used to “zoom into” the towns and regions of students’ countries of origin. Students can adopt a comparative approach to compare aspects of their countries of origin to Canadian realities that are incorporated into the curriculum expectations of the social studies curriculum (see Cummins and Persad, in press, for examples). Obviously, parents can participate in this process by describing aspects of the culture and landscape and supplying additional artifacts.
• Students’ languages can be integrated in creative ways into a variety of content instruction. For example, Grade 5 teacher, Tobin Zikmanis, in the Peel District School Board addressed the Ministry curriculum expectations in the Data Management Unit of the Math curriculum by having students carry out a language survey of the entire school and then using spreadsheet software to generate a variety of graphs (e.g., pie charts, bar graphs) to display and disseminate their findings.

### DUAL LANGUAGE PROJECT WORK

• Students can write and web-publish dual language stories or projects (see Lotherington, 2011 for examples and also^3^^,^^4^). Where students are learning French, the book or project production can be trilingual (L1, English, French). An excellent resource for facilitating the web-publication of multilingual books is the website and iPad application *Scribjab* ^5^ developed by Simon Fraser University researchers Dr. Diane Dagenais and Dr. Kelleen Toohey. Scribjab allows students to upload their creative writing and audio recordings of this writing and to read and listen to other students’ stories^6^.
• Students can collaborate with partner classes in distant locations (across the world or across the city) to carry out a variety of projects involving dual or multiple languages. These projects could focus on social justice issues (e.g., environmental policies, income disparities, etc. in different countries) or other substantive curriculum-relevant content.

These examples are illustrative of the pedagogical options that open up when educators adopt a multilingual lens. Many other examples of “translanguaging” have been described in the publication *Translanguaging* (Celic and Seltzer, 2011; available for free download at^7^). Dr. Roma Chumak-Horbatsch (2012) of Ryerson University has also documented a wide variety of multilingual instructional activities for early childhood education and primary grades in her book *Linguistically Appropriate Practice* (see also her website at^8^).

## CONCLUSION

The critique of Canadian educational provision in relation to language development issues in this paper is not in any sense intended to undermine the commitment to quality education that educators and policy-makers alike have pursued over several decades. Canadian education has generally avoided the dysfunctional ideological battles that have characterized education in the United States during this period (e.g., in relation to reading instruction, bilingual education, school funding, etc.). Achievement outcomes of Canadian education also compare well with those of other countries (e.g., OECD, 2010).

However, Canadian policy-makers have not responded adequately to the instructional challenges and opportunities afforded by Canadian multilingual realities. With respect to the education of immigrant-background students, we have failed to ensure that Canadian school administrators and educators in mainstream classrooms have had opportunities and incentives to develop the instructional expertise to teach these students effectively. For more than 40 years of consistently high levels of immigration to Canada since the early 1970s, Faculties of Education in English Canada have viewed the job of teaching DLL students as the job of the specialist ESL teacher. There are indications of a change in thinking in relation to this issue in some provinces but there is still a long way to go before the well-worn mantra of “capacity-building” is extended to include building the capacity of all teachers and administrators to educate DLL students in an evidence-based way.

Similarly, with the notable exception of the province of Alberta, there has been a policy vacuum with respect to imaginative educational responses to Canada’s multilingual resources. For the most part, we have been content to stand on the sidelines as observers while children’s home languages slip away from them in the early years of schooling. The exercise in imaginative thinking that generated French immersion programs as well as more recent initiatives such as Intensive French and AIM, has been largely stifled by restrictive provincial policies and administrative inertia that continue to frustrate parents and community members who attempt to initiate effective programs for the teaching of languages other than English and French.

On a positive note, the seeds of educational change have been planted in cities across Canada by educators in individual schools who have not waited either for community pressure or top-down mandates to implement instruction that is truly imaginative and inspirational. School/university collaborations in communities across Canada have articulated and field-tested imaginative instructional strategies that build language awareness and proactively communicate to students that their multilingual abilities contribute significantly to their own identities, their communication with family members, and to the cultural richness of their school communities. The commitment of these educators to repudiate the notion of the school as an English-only zone (or French-only zone in Quebec) in favor of teaching through a multilingual lens is identity-affirming both for them as educators and for their students whose intellectual, cultural, and linguistic resources are being constructed rather than constricted by their educational experiences. The challenge of the next decade is to scale up these initiatives so that they become institutionalized as educational policy rather than just the inspired teaching of exceptional educators.

## Conflict of Interest Statement

The author declares that the research was conducted in the absence of any commercial or financial relationships that could be construed as a potential conflict of interest.

## Footnotes

1 http://www.caslt.org/pdf/aim.pdf

2 http://www.translate.google.com

3 http://schools.peelschools.org/1363/pages/dual.aspx

4 http://www.multiliteracies.ca/index.php/folio/viewProject/8

5 www.scribjab.com

6 http://www.sfu.ca/education/newsevents/foe-news/2014/january-2014/diane-dagenais-speaks-to-radio-canada-about-scribjab.html

7 http://www.nysieb.ws.gc.cuny.edu/publicationsresources/

8 http://www.mylanguage.ca
